# Biological Pathways Involved in Tumor Angiogenesis and Bevacizumab Based Anti-Angiogenic Therapy with Special References to Ovarian Cancer

**DOI:** 10.3390/ijms18091967

**Published:** 2017-09-14

**Authors:** Vera Loizzi, Vittoria Del Vecchio, Giulio Gargano, Maria De Liso, Anila Kardashi, Emanuele Naglieri, Leonardo Resta, Ettore Cicinelli, Gennaro Cormio

**Affiliations:** 1 Department of Biomedical Sciences and Human Oncology, University of Bari, 70121 Bari, Italy; vittoria.delvecchiomd@gmail.com (V.D.V.); ettore.cicinelli@uniba.it (E.C.); gennaro.cormio@uniba.it (G.C.); 2 Gynecologic Oncology Unit, IRCCS, Istituto Tumori Giovanni Paolo II, 70142 Bari, Italy; g.gargano@oncologico.bari.it (G.G.); m.deliso@oncologico.bari.it (M.D.L.); a.kardhashi@oncologico.bari.it (A.K.); emanuele.naglieri@gmail.com (E.N.); 3 Department of Pathology, University of Bari, 70121 Bari, Italy; leonardo.resta@uniba.it

**Keywords:** angiogenesis, VEGF, ovarian cancer

## Abstract

The creation of new blood vessels from existing ones, which is a mechanism called “angiogenesis”, is essential in cancer to supply cancerous growth. Moreover, the development and the progression of the tumor and its metastases are the result of an efficient vascular response. Cancer cells release and activate different angiogenic growth factors and their receptors in the tumor microenvironment to promote the angiogenic process. The most important pro-angiogenic factor is the “Vascular Endothelial Growth Factor” (VEGF) because of its mitogen activity on vascular endothelium. Bevacizumab is a monoclonal antibody that obstructs the binding of circulating vascular endothelial growth factor to its receptors and has been approved for the treatment of primary and recurrent ovarian cancer but also for many other solid tumors.

## 1. Introduction

Tumors are composed of cancerous cells and stroma, which consists in fibroblasts, macrophages, mast cells, vascular and lymphoid endothelium, perycites, cells for the immunity response, nerves and extracellular matrix proteins. The biological behavior of tumors depends on the context in which cancer cells exist [1].

**Figure 1:**
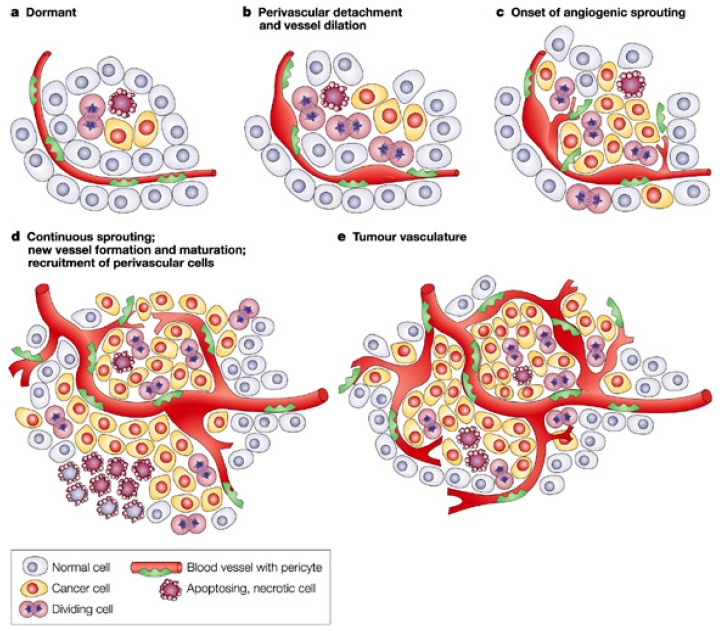
(**a**–**e**) Angiogenic “switch” from dormant cells to tumor vasculature. Adapted from Bergers, G. Benjamin, L.E. *Nat. Rev. Cancer* **2003**, *3*, 401. Copyright 2003 Nature Publishing Group.

In cancer, angiogenesis is the mechanism that permits the creation of new blood vessels from existing ones to supply cancerous growth. The development and the progression of the tumor and its metastases are the result of an efficient vascular response [2]. The beginning of this process is known as “angiogenic switch”, through which tumors acquire the ability to grow and disseminate beyond their primary site [3], and which can be activated by hypoxia, hypoglycemia, mechanical stress and inflammation (Figure 1). The switch consists in the following different steps: perivascular detachment and vessel dilation, angiogenic sprouting, new vessel creation and development, and recruitment of perivascular cells. New blood vessels will support tumor growth, specifically feeding hypoxic and necrotic areas of the tumor to provide it with essential nutrients and oxygen [4].

Tumor cells release molecules that induce the surrounding normal host tissue to promote the growth of new blood vessels, letting the angiogenesis process begin [2].

Together with this mechanism, which is the best-known one, other mechanisms are involved in promoting angiogenesis in cancer: the vessel co-option, the intussusceptive microvascular growth, the glomeruloid angiogenesis, the postnatal vasculogenesis and the so-called vasculogenic mimicry. In the vessel co-option, cancer cells come into contact with host surface of pre-existing blood vessels and grow along them, thanks to the incorporation of host tissue capillaries; the insussusceptive microvascular growth consists of the formation of new vessels from connective tissue pillars that grow in the lumen of blood vessels, as a consequence of the stimulus of different cytokines such as platelet-derived growth Factor (PDGF), transforming growth factor beta (TGF-β) and agiopoietins; glomeruloid angiogenesis starts from the so-called glomeruloid bodies that are composed by host vascular aggregates, basement membrane and pericytes and that appear as a consequence of the extravasation of cancer cells; postnatal vasculogenesis consists of the transformation of the endothelial progenitor cells derived from bone marrow in endothelial cells, as a consequence of the stimulation of pro-angiogenic factors such as VEGF and PDGF that adhere on the surrounding host mature vessels and promote the formation of new ones; and, finally, vasculogenic mimicry consists of cancer cells behaving as endothelial cells producing vessel-like structures that provide blood to the tumor [5].

Under physiological conditions, angiogenesis is a consequence of ischemic and hypoxic signals; during tumor angiogenesis, the angiogenesis process is uncontrolled and upregulated because of a predominance of pro-angiogenic factors compared with anti-angiogenic ones.

**Figure 2:**
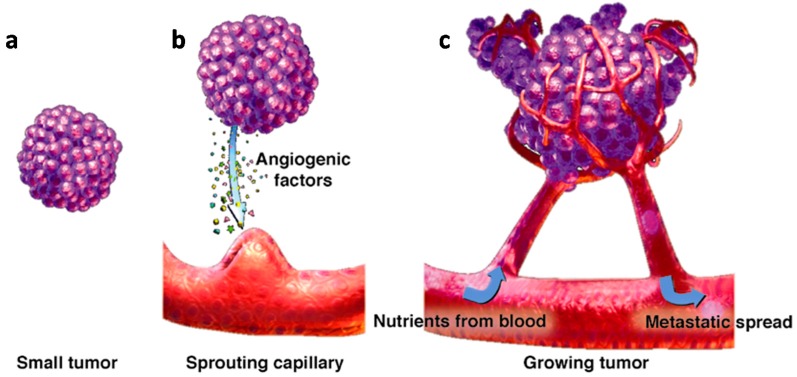
(**a–c**) Tumor expansion induced by the sprouting of blood vessels. From small tumor (**a**), sprouting capillary (**b**) to growing tumor (**c**). Adapted from Garrel, D.; Augstburger, R.; *Townsend Lett. Dr. Patients* **2004**, *246*, 74–79. Copyright 2004 Townsend Letter.

The hypoxic microenvironment activates the angiogenic network promoting the sprouting of new blood vessels into the tumor and inducing its mass expansion (Figure 2).

Abnormal angiogenesis is characterized by the increasing of proliferation of endothelial cells, by atypical morphology of the tumor vasculature, of endothelial cells and pericytes, smooth muscle cells and of the basement membrane [3]. Compared to normal blood vessels, the tumor ones are characterized by high vascular permeability, poor blood flow and irregular shape [6].

Angiogenic growth factors and their corresponding receptors, which are released and activated by cancer cells, are the main responsible of the start of the angiogenic process.

## 2. Main Pro-Angiogenic Factors

Ovarian cancer cells both express and respond to many types of growth factors that bind to cell surface receptors and activate signaling pathways that promote the activation or the inhibition of specific genes. In cancer cells, these pathways are commonly dysregulated, inducing continuous signaling (Table 1).

### 2.1. Vascular Endothelial Growth Factor (VEGF)

Induced by hypoxia, it regulates new vessels growth. Transcription of VEGF mRNA is mediated by Hypoxia inducible factor-1 (HIF-1) which also promotes downregulation of anti-angiogenic factors such as trombospondin-1 and angiostatin [7]. Secretion of VEGF is also promoted by hormones (estrogen and progesterone) and cytokines such as EGF, TNF, IL-1, IL-6, PDGF and prostaglandins. VEGF-family is composed by seven members, VEGF A–E, and placental growth factor (PIGF) 1 and 2 [6]. These molecules exert their effect binding to their tyrosine kinase receptors normally expressed on the surface of endothelial cells which are made of an extracellular region composed by seven immunoglobuline-like domain, a transmembrane region and an intracellular one that has the tyrosine kinase activity [2]. The main actions of VEGF are: induction of angiogenesis; stimulation of growth and proliferation of endothelial cells, and inhibition of their apoptosis; activation of enzymes involved in extracellular matrix degradation; promotion of mobilization of bone marrow endothelial precursors; and regulation of vascular permeability [2].

VEGF levels in ovarian cancer are associated with advanced tumor stage, onset of metastases, poor progression free survival and overall survival. The overexpression of VEGF can transform normal ovarian epithelial cells into ascites-producing cells and neoplastic ones. Moreover, in malignant ovarian tumors, VEGF overexpression is correlated with the development of ascites, carcinomatosis and poor prognosis [7].

### 2.2. Platelet Derived Growth Factor (PDGF)

It is involved in vessels maturation and recruitment of pericytes through the four isoforms PDGF A–D.

PDGF is secreted by activated platelets, endothelial, epithelial, glial and inflammatory cells. Through paracrine stimulation, this cytokine induces neighboring cells to proliferate and migrate in many physiological processes. Different types of genetic alterations in tumors can activate PDGF ligands and receptors that act as proto-oncogenes. PDGF receptors (PDGFRα and β), which are expressed in oligodendrocytes, fibroblasts and vascular smooth muscle cells, are receptor tyrosine kinases that, in the active forms, consist of two chains associated with one dimeric ligand. PDGFRα binds to PDGFA-C isoforms; and PDGFRβ is activated by PDGF-B and -D.

Together with the promotion of proliferation of cancer cells, PDGF can also induce angiogenesis and the creation of the tumor associated fibroblasts in different types of tumors [8].

In malignant ovarian tumors, PDGF is overexpressed and has been dosed in a large number of samples, together with its receptors, which are present in malignant ascites too [6]. Moreover, patients with ovarian cancer expressing the PDGF receptor demonstrate an overall shorter survival time compared with those whose tumors did not express the receptor [9]. PDGFA and -B have been found in serum of patients with advanced International Federation of Gynecology and Obstetrics stages (III–IV).

### 2.3. Fibroblast Growth Factor (FGF)

It includes secreted proteins (secreted FGFs) that bind to receptor tyrosine kinases. In adult, secreted FGFs act as homeostatic factors implicated in tissue maintenance, repair, regrowth and metabolism. Secreted FGFs regulate metabolism as well as proliferation of cells, and their survival, migration and differentiation. FGF signaling pathway involves different proteins such as mitogen-activated protein kinases (MAPK) and proteins of the PI3k/Akt cascade [3]. Abnormalities involving the structure of FGF receptor (FGFR) can provoke anomalous morphogenesis and the development of different types of cancer [10]. FGF also promotes angiogenesis acting together with VEGF and other pro-angiogenic factors. Over 20 isoforms have been identified that link to five types of receptors. The first four isoforms of the receptors consist in an extracellular immunoglobulin-like domain and an intracellular tyrosine kinase one. In the fifth isoform, the intracellular tyrosine kinase domain is missing. Aberrations of FGF signaling pathways have been found in different types of human and animal cancers; FGF overexpression and gene amplification promote initiation and progression of tumors.

In malignant ovarian tumors, FGF’s expression may be associated with prognosis, cancer progression and new blood vessel creation, and it has been found in ascites together with VEGF [6]; moreover, FGFR1, -3, and -4 have been amplified and demonstrated in ovarian tumors.

### 2.4. Epidermal Growth Factor (EGF)

It is involved in tumors arising from epithelial cells inducing cancer growth, dissemination, invasion and metastases. EGF receptor (EGFR) belongs to ErbB family of tyrosine kinase receptors (RTKs) that induce cells stimulated by an EGFR ligand to grow and proliferate. EGFR is formed by an extracellular and a transmembrane domain, a juta membrane domain, the intracellular domain with the tyrosine kinase action and a C-terminal tail with many tyrosine residues [7]. In cancer, EGFR is often mutated and, for this reason, continually activated and stimulated because of the sustained production of EGFR ligands such as EGF, TGF*α*, amphiregulina and heparin-binding EGF. Anomalous expression of TGF*α* or EGFR by cancer cells is associated with an aggressive phenotype and poor prognosis [11], also because the activation of EGFR signaling results in up-regulation of pro-angiogenic factors such as VEGF.

In malignant ovarian tumors, cancer cells growth and survival is induced by autocrine and paracrine stimulation of EGFR and its overexpression and hyperactivity is associated with resistance to anticancer treatments; moreover, activation of the EGFR receptor is associated with sprouting of metastases [12].

### 2.5. Transforming Growth Factor (TGF)

It acts as pro-angiogenic factor when its levels are low by the up-regulation of angiogenic factors and proteases, and as anti-angiogenic factor when its levels are high by the inhibition of endothelial cells’ growth and proliferation [2].

In mammals, three isoforms of TGF-β (TGF-β1, -β2 and -β3) exist in a latent form, requiring activation before they can exert biological activity, and bind to two serine/threonine kinases membrane receptors (type I and type II). TGF-β is at least partly responsible for activation of fibroblasts in cases of a number of different cancer types. The tumor microenvironment, mainly composed of fibroblasts, proteins, endothelial cells and lymphocytic infiltrates, promotes cell growth, migration and differentiation through the action of secreted proteins, cell–cell interactions, and matrix remodeling. In ovarian cancer, tumor growth may be promoted by TGF-β which regulates the secretion of stroma-specific mediators in the tumor microenvironment. It enhances the spreading of ovarian cancer cells by upregulating versican gene (VCAN) in cancer-associated fibroblasts (CAFs), through the activation of type II receptor. When VCAN gene is upregulated, NF-κB signaling pathway is activated and promotes motility and invasion of ovarian cancer cells [10]. On the surface of ovarian cancer cells lower levels of both TGF-β have been found, compared with the surface of normal ovarian epithelial cells, suggesting that resistance to TGF-β in ovarian cancer cells could be induced by downregulation of the receptors [13]. BMPs (bone morphogenetic proteins) are cytokines belonging to the family of TGF-β and are responsible of the development and progression of cancer, depending on the characteristics of the microenvironment in which tumor grows, and of metastatic spread. They act promoting angiogenesis and the inhibition of apoptosis of cancer cells through two types of serine/threonine receptors (type I and type II), activating SMAD signaling pathway. In ovarian cancer, overexpression of BMP-2, BMP-4 and BMP-7 has been reported [14].

### 2.6. Matrix Metalloproteinases (MMPs)

Through degradation of the extracellular matrix and the emission of angiogenic mitogens, they induce tumor angiogenesis. MMP-9 and MMP-2 promote neo-angiogenesis in cancer proteolitically cleaving and activating latent TGF-β [2].

MMP family is composed by 23 members and many of them are associated with ovarian cancer. MMPs are induced by ovarian cancer cells and by extracellular matrix to promote tumor growth, invasion and dissemination. MMPs act on different extracellular matrix (ECM) components such as collagens, gelatins, fibronectins and laminins, leading to changes in its structure and the expression of its cellular surface receptors promoting occurrence, development, invasion and metastases of malignant tumors, and may be the main responsible of the disruption of the balance between growth and antigrowth signals. In ovarian cancer, MMPs’ overexpression is associated with tumor cells dissemination and metastases, with poor prognosis and decreased survival [15]. MMP-1 is overexpressed in different malignant tumors and is associated with invasion of epithelial ovarian cancer cells, lymph-node involvement and metastases [16].

### 2.7. Tumor Necrosis Factor (TNF)

Released by macrophage, mast cells and T-lymphocytes, it is a cytokine that activates macrophages and induces the releasing of angiogenic factors [2]. It can act differently, inducing apoptosis, angiogenesis, necrosis, cell migration, immune cell activation and differentiation, depending on the cellular background, which are of great relevance in tumor immune surveillance, in tumor development and progression [17]. Epidemiologic studies identified in the development of ovarian cancer an important inflammatory stimulus. Acting by an autocrine way, TNF-α promotes the releasing and the activation of other cytokines and angiogenic factors that promote the diffusion of cancer to the peritoneum and the angiogenic process [18]. Indeed, in biopsies of this malignancy, high levels of the pro-inflammatory cytokine TNF-α and of both its receptors (TNFRI and TNFRII) are expressed and are associated with increased tumor grade.

### 2.8. Angiopoietins

Angiopoietin 1 has both agonist and antagonist activities, acting as pro-angiogenic and anti-angiogenic factor through Tie receptors and its signaling pathway [2]. Ang-1 and -2, interacting with the tyrosine kinase receptor Tie2, mainly expressed on the vascular endothelium, can promote neo-angiogenesis, instead Ang-1 acts through the Akt/surviving pathway stabilizing newly produced vessels. Moreover, to fix and promote vasculature, Ang-2, which is mainly expressed in endothelial cells and tumor stroma, may act alone or together with other pro-angiogenic factors, such as VEGF, also correlating with their expression levels [6], promoting stabilization and maturation of new vessels.

Compared with normal ovary, expression of Ang-1, Ang-2 and Ang-4 is elevated in primary serous carcinoma as well as in omental metastatic lesions of serous carcinoma, one of the most common sites of ovarian cancer metastasis. There are distinct localization patterns of angiopoietins within the tumor: Ang-1 and Ang-2 are more concentrated in the tumor cells, whereas Ang-4 has a higher expression level in the tumor stroma. This pattern is consistent in both primary and metastatic lesions [19].

## 3. Bevacizumab: Structure Activity Data

Angiogenesis is an important factor in the cancerogenesis, growth and progression of human hematological and solid tumors [20]. In fact, studies with animals suggest that angiogenesis is an important factor in tumor development [21,22]. Among the pro-angiogenic factors, vascular endothelial growth factor (VEGF), known as vascular permeability factor, results the most important as a mitogen for vascular endothelium [23]. Recombinant humanized mAb (RhumAb) anti-VEGF that is known as bevacizumab (the RhumAb; AvastinTM; Genentech, Inc.; South San Francisco, CA, USA) is a RhumAb composed of the consensus human IgG1 framework and antigen-binding regions (93%) and compliment-determining regions from the murine mAb A.4.6.1 (7%) [24].

The role of bevacizumab is to neutralize all isoforms of human VEGF with a dissociation constant (kd) of 1.1 and 0.8 nmol/L, respectively. In addition, it inhibits VEGF-induced proliferation of endothelial cells in vitro with an ED_50_ of 50 ± 5 and 48 ± 8 ng/mL. The distribution analyzed in a rabbit model has demonstrated that the majority of bevacizumab persisted in the plasma, with a large quantity being distributed to the bladder, kidney, heart and testes, as compared to other organs. Data have also suggested that bevacizumab is cleared from the circulation in a manner similar to that observed for endogenous antibodies [25]. The terminal elimination half-life of bevacizumab is about 1–2 weeks in all species, and 125 iodine antibody localization studies indicate that it is distributed to areas that are highly perfused (albeit with minimal localization to the liver) with a linear pharmacokinetic profile [26]. Studies in mice, rats and rabbits indicate that it would be necessary to have plasma bevacizumab concentrations of about 10–30 g/mL to obtain maximal tumor growth inhibition. Doses of 0.3 mg/kg gave complete suppression of free serum VEGF, whereas doses of greater than 1 mg/kg produced serum levels of bevacizumab in the target range of 10 g/mL for at least 14 days [27].

## 4. Bevacizumab in Ovarian Cancer

The standard treatment for epithelial ovarian cancer (EOC) consists of optimal cytoreductive surgery followed by platinum-based chemotherapy [28]. Bevacizumab has been approved for the treatment of metastatic colorectal cancer and advanced renal cell carcinoma, and as first-line treatment for advanced non-squamous non-small cell lung cancer. In addition, it is approved as first- and second-line treatment for advanced epithelial ovarian, fallopian tube, and primary peritoneal cancers. The reason of its use in ovarian cancer is primarily based on the results of randomized clinical trials showing improved survival rates with the addition of bevacizumab to standard first-line chemotherapy, mainly in patients at high-risk [29]. However, positive results have been observed in second-line treatment, in both platinum sensitive and platinum-resistant disease [30].

Even if the standard treatment for epithelial ovarian cancer (EOC) consists of optimal cytoreductive surgery followed by platinum-based chemotherapy [28,31], interval debulking surgery (IDS) after a short course of neoadjuvant chemotherapy (NACT) could represent a valid alternative for patients who are unable to undergo upfront complete resection. Some studies showed no significant differences in progression-free survival (PFS) in EOC patients undergoing IDS compared to those treated with primary debulking surgery. However, fewer adverse effects and lower mortality rates were seen in the IDS group [32,33].

### 4.1. Neoadjuvant Setting

Few studies [34,35] have been conducted with bevacizumab in the neoadjuvant setting for advanced OC. The results are controversial, however the use of bevacizumab is not yet recommended in this setting because of wound complications, gastrointestinal (GI) perforations, fistulas, and thromboembolic incidents [36]. We expect that confirmation of the efficacy and safety of bevacizumab will arrive from the Anthalya study, a French ongoing, randomized, phase II trial that involved patients with initially unresectable FIGO stage IIIC/IV ovarian, tubal, or peritoneal adenocarcinoma [37].

### 4.2. First Line Setting

The efficacy of bevacizumab in addition to carboplatin and paclitaxel as first-line treatment in OC patients was demonstrated in two randomized multicenter trials: GOG-218 and the ICON7 [29,38]. The first one [29] included patients with FIGO stage III–IV advanced epithelial ovarian, fallopian tube, or peritoneal cancer. The patients received a first-line chemotherapeutic treatment as follows: the first group three-weekly cycles of carboplatin and paclitaxel in Cycles 1–6; the second group the same chemotherapy with concomitant bevacizumab in Cycles 2–6 and placebo in Cycles 7–22; and the third group the same chemotherapy plus bevacizumab in Cycles 2–22. The results of the study revealed a significantly increase in PFS in patients receiving bevacizumab plus carboplatin and paclitaxel followed by maintenance bevacizumab with respect to patients treated only with standard chemotherapy or without bevacizumab maintenance (14.1 vs. 10.3 and 11.2 months, respectively, *p* < 0.001). However, they also showed that the addition of bevacizumab to standard chemotherapy did not significantly improve the OS.

The ICON 7 was the second phase III randomized trial that showed similar results. Patients with FIGO stage I–IIA (clear-cell histology or grade 3) and IIB–IV EOC, carcinoma of the fallopian tubes, or peritoneal cancer were included. All patients had six cycles of three-weekly carboplatin (AUC 5 or 6) and paclitaxel 175 mg/m^2^ with or without bevacizumab (7.5 mg/kg) for 12 months [38]. The primary endpoint was PFS which showed to be significant difference between the two groups. In fact the median PFS was 17.4 months in the controls and 19.8 months in the group that had the addition of bevacizumab (*p* = 0.0041). Similarly, the overall responses were significantly different with an overall response of 48% in the chemotherapy-alone group and 67% in the bevacizumab group, respectively (*p* < 0.0001). No differences in the OS rate were reported.

### 4.3. Recurrent EOC Setting

Platinum-sensitive OC patient is defined who has a progression-free interval greater than six months from the last platinum chemotherapy. For this reason, patients who relapse within this interval are usually treated with platinum-based therapy and carboplatin is typically used in combination with paclitaxel or with pegylated liposomal doxorubicin (PLD), or gemcitabine [39,40,41], achieving a mild benefit in terms of progression free survival (PFS) and overall survival (OS). The two trials that investigated the role of bevacizumab in platinum-sensitive recurrent OC were: the OCEANS and the GOG-213 trials. The first is a randomized, double-blind, placebo-controlled, phase III trial that enrolled patients to receive the standard chemotherapy based on intravenous gemcitabine plus carboplatin and placebo or the standard chemotherapy plus 3-weekly bevacizumab (15 mg/kg) until disease progression or unacceptable toxicity. At a median follow-up of 24 months, the median PFS was significantly longer in the group who were treated with bevacizumab than in the placebo group (12.4 vs. 8.4 months, respectively, *p* < 0.001) [42]. The second trial is the Gynecologic Oncology Group (GOG)-0213. This is a phase III randomized, double-bind, placebo-controlled study conducted in patients affected by platinum-sensitive EOC or fallopian tube or peritoneal cancer. Even in this trial, the results confirmed an increase in PFS of 3.4 months in the group including bevacizumab. In this study, an increase in OS was also observed (*p* = 0.056) [43].

Platinum-resistant OC patient is defined who has a progression-free interval less than six months from the last platinum chemotherapy. Several new cytotoxic drugs have been assessed in chemoresistant EOC, such as bevacizumab. Patients who had platinum resistant disease have a poor outcome with a lower tumor response rates. The median OS of these patients is of 9–12 months [44,45]. Only one randomized, open-label, phase III, multicenter study has evaluated the efficacy and tolerability of adding bevacizumab to single-agent chemotherapy in patients with recurrent, platinum-resistant epithelial ovarian, fallopian tube, or primary peritoneal cancer. Patients received single-agent chemotherapy (paclitaxel, PLD, or topotecan) alone or in combination with bevacizumab until disease progression or unacceptable toxicity (the AURELIA study). In this study, a significant benefit was observed in PFS in patients receiving bevacizumab plus chemotherapy (6.7 months) than in those receiving chemotherapy alone (3.4 months, *p* = 0.001). However, the OS showed no significant difference. An increase by more than 15% of abdominal/GI symptoms in patients with recurrent, platinum-resistant disease was observed with the addition of bevacizumab to the chemotherapy [46]. However, to delineate a definitive profile of efficacy and tolerability of bevacizumab in the setting of “platinum-resistant” OC patients, the results of ongoing trials investigating the role of bevacizumab alone or in combination with conventional anti-blastic agents are necessary.

## 5. Conclusions

Bevacizumab is a humanized monoclonal antibody. Its role is to block the binding of circulating vascular endothelial growth factor to its receptors. To date, bevacizumab has been approved for the treatment of several solid tumors, including OC in both first and second-line.

## Figures and Tables

**Table 1:** Pro-angiogenic factors, molecular weight and receptors.

Pro-Angiogenic Factors	Molecular Weight	Receptors
VEGF	19 kDa	Tyrosine kinase receptors (VEGFR1, VEGFR2 and VEGFR3)
PDFG	30 kDa	Tyrosine kinase receptors (PDGFRα and β)
FGF	18 kDa	Tyrosine kinase receptors (FGFR1, FGFR2, FGFR3, and FGFR4)
EGF	6.4 kDa	Tyrosine kinase receptors: EGFR (ErbB1, HER1), ErbB2 (HER2), ErbB3 (HER3) and ErbB4 (HER4)
TGF	25 kDa	Serine/threonine kinase receptors (type I and type II)
MMP’S	125 kDa	Low-density lipoprotein receptor-related protein (LRP)
TNF	51 kDa	Tyrosine kinase receptors (TNFRI and TNFRII)
ANGIOPOIETIN	57 kDa	Tyrosine kinase receptors (Tie-1 and Tie-2)

Vascular Endothelial Growth Factor (VEGF); Platelet Derived Growth Factor (PDGF); Fibroblast Growth Factor (FGF); Epidermal Growth Factor (EGF); Transforming Growth Factor (TGF); Matrix metalloproteinases (MMPs); Tumor Necrosis Factor (TNF) and Angiopoietins [2].
